# Editorial: Recent advances in agricultural waste recycling by microorganisms and their symbiosis

**DOI:** 10.3389/fmicb.2025.1631828

**Published:** 2025-06-10

**Authors:** Qi Zhang, Xiang Wang, Pengfei Cheng, Shuhao Huo, Cuixia Liu, Zhigang Yu

**Affiliations:** ^1^State Key Laboratory of Food Science and Resources, Engineering Research Center for Biomass Conversion, Ministry of Education, Nanchang University, Nanchang, Jiangxi, China; ^2^Key Laboratory of Eutrophication and Red Tide Prevention of Guangdong Higher Education Institutes, College of Life Science and Technology, Jinan University, Guangzhou, China; ^3^College of Food and Pharmaceutical Sciences, Ningbo University, Ningbo, Zhejiang, China; ^4^School of Food and Biological Engineering, Jiangsu University, Zhenjiang, China; ^5^School of Energy & Environment, Zhongyuan University of Technology, Zhengzhou, China; ^6^Australian Centre for Water and Environmental Biotechnology (formerly AWMC), The University of Queensland, Brisbane, QLD, Australia

**Keywords:** microalgae, wastewater treatment, resource utilization, microbial-algal symbiosis, metagenomics, transcriptomics, metabolomics, synthetic biology

## 1 Introduction

The mounting environmental burden caused by agricultural waste has drawn increasing global concern. From crop residues and livestock manure to agro-industrial effluents, these waste streams are rich in organic matter and nutrients, yet often underutilized or improperly managed. Microorganisms-particularly microalgae, bacteria, and fungi-have emerged as powerful agents in transforming such waste into value-added products, offering sustainable alternatives to conventional waste treatment and resource recovery strategies. Within this context, our Research Topic was conceived to showcase emerging trends, collaborative approaches, and integrative biotechnologies driving the frontier of microbial waste recycling.

The published contributions in this Research Topic reflect a multifaceted research landscape that integrates applied microbiology, environmental engineering, and systems biology. Together, they illustrate how microbial consortia, metabolic regulation, and bioreactor optimization converge to unlock the potential of agricultural waste. Rather than presenting a mere listing of articles, this editorial highlights how these studies contribute to broader scientific and societal goals, including sustainable development, circular bioeconomy, and pollution control.

## 2 Overview of contributions

This Research Topic received and successfully published 11 peer-reviewed articles from authors across Asia, Europe, and Oceania. These studies exemplify recent scientific and technological advances in microbial-driven valorization of agricultural waste.

Several studies demonstrate the innovative use of microbial consortia to enhance composting efficiency and nutrient retention. For example, Wang et al. reported the use of thermotolerant strains to accelerate manure composting, providing insights into thermophilic microbial ecology under high-temperature conditions. Xie C. et al. expanded this by integrating tobacco waste into compost substrates, enhancing nitrogen preservation and seed germination-practical outcomes for soil fertility and sustainable agriculture.

Straw and lignocellulosic waste remain difficult substrates for bioconversion due to their recalcitrant structure. Ma et al. addressed this by employing *Rhodococcus wratislaviensis* YZ02 to achieve high cellulase activity and effective degradation under optimized fermentation conditions, pointing to the promise of actinobacteria in lignocellulose valorization. Complementary work by Yu et al. explored white-rot fungi and indigenous bacteria in a co-cultivation system, enhancing lignin degradation and producing protein-rich biomass suitable for ruminant feed. Chen et al. developed a multi-strain probiotic and enzyme synergistic fermentation system to convert distillers' grains into nutrient-rich animal feed, addressing both waste reduction and feed security. Xie Y. et al. performed solid-state fermentation of brewers' spent grain and tracked microbial succession and metabolite changes, offering insights for feed safety and compositional stability.

Symbiotic interactions among microalgae and bacteria are also gaining traction for their dual roles in pollutant removal and biomass production. Zheng, Liu, et al. designed an inverse fluidized bed bioreactor where algae-bacteria symbiosis achieved over 95% nitrogen removal without external aeration, indicating energy-efficient solutions for wastewater polishing. Zheng, Zhang, et al. also investigated microbial community evolution during para-nitrophenol (PNP) removal in an aerobic granular sludge system, enhancing our understanding of microbial pollutant degradation. Similarly, Mingcheng et al. leveraged microalgae for ammonia reduction, elucidating microbial functional pathways via metagenomic and transcriptomic profiling.

Advanced microbial detection and optimization techniques underpin several studies. Xie Y. et al. used high-throughput sequencing to monitor microbial succession during composting, offering predictive models for process control. Hao et al. studied the stress responses of diatoms under dehydration conditions, contributing to the theoretical foundation for applying microalgae in arid environment biotechnology. Zheng Y. et al. conducted a complete genome analysis of a *Bacillus velezensis* strain with broad-spectrum antagonistic activity, providing molecular evidence for its potential use in biocontrol and organic waste stabilization.

## 3 Conclusion

Taken together, the 11 articles featured in this Research Topic transcend traditional disciplinary boundaries. They collectively advocate for integrated microbiological approaches to agricultural waste management—highlighting not only innovations in laboratory settings, but also practical, scalable solutions for the field. These works underscore a shift toward ecosystem-inspired technologies, metabolic network engineering, and multi-functional microbial systems tailored to real-world complexity.

This Research Topic serves as both a benchmark and a catalyst for future research that integrates microbial ecology, biotechnology, and systems engineering in the pursuit of a more sustainable agricultural future. We thank all contributing authors, reviewers, and the Frontiers editorial team for their invaluable support and look forward to ongoing developments in this critical field.

